# Who is at risk of myopia?

**Published:** 2019-05-13

**Authors:** Tim Fricke, Priya Morjaria, Padmaja Sankaridurg

Ethnicity is a significant risk factor, with individuals from East and South East Asian countries at greater risk of developing myopia.^1^ More importantly, myopia begins at an earlier age in these individuals, resulting in a greater number of years during which myopia can progress. This increases the risk that they will develop high myopia.^2^

Older children are more likely to develop myopia.^3^ However, annual progression is significantly greater in younger children.^4^ A 6-year old child with myopia will have significantly greater progression than, for example, a 10-year-old, placing them at greater risk of high myopia.

Parental myopia may also influence onset with those with both parents being myopic at greater risk of developing myopia.^2^

There is also a small difference in prevalence between males and females, with females at greater risk of myopia than males.^5^

**Figure:**
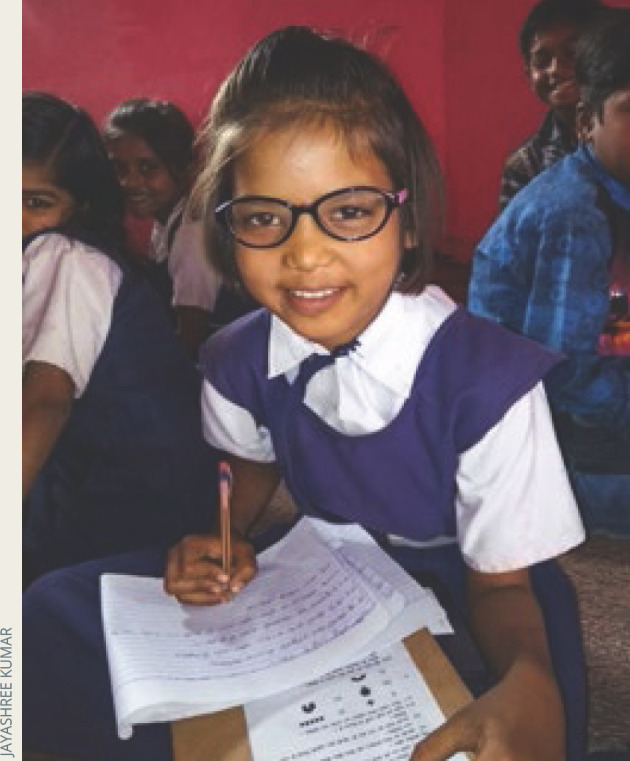
Girls are more likely than boys to develop myopia, although the difference is small. INDIA
